# Cortical–subcortical interactions underlie processing of auditory predictions measured with 7T fMRI

**DOI:** 10.1093/cercor/bhae316

**Published:** 2024-08-01

**Authors:** Alberto Ara, Vasiliki Provias, Kevin Sitek, Emily B J Coffey, Robert J Zatorre

**Affiliations:** Montreal Neurological Institute, McGill University, 3801 University Street, Montreal, QC H3A 2B4, Canada; International Laboratory for Brain, Music and Sound Research (BRAMS), 90 Vincent-d’Indy Avenue, Outremont, QC H2V 2S9, Canada; Centre for Research in Brain, Language and Music (CRBLM), 3640 de la Montagne Street, Montreal, QC H3G 2A8, Canada; International Laboratory for Brain, Music and Sound Research (BRAMS), 90 Vincent-d’Indy Avenue, Outremont, QC H2V 2S9, Canada; Centre for Research in Brain, Language and Music (CRBLM), 3640 de la Montagne Street, Montreal, QC H3G 2A8, Canada; Department of Psychology, Concordia University, 7141 Sherbrooke Street West, Montreal, QCH4B 1R6, Canada; Department of Communication Sciences and Disorders, Northwestern University, 2240 Campus Drive, Evanston, 60208 IL, USA; International Laboratory for Brain, Music and Sound Research (BRAMS), 90 Vincent-d’Indy Avenue, Outremont, QC H2V 2S9, Canada; Centre for Research in Brain, Language and Music (CRBLM), 3640 de la Montagne Street, Montreal, QC H3G 2A8, Canada; Department of Psychology, Concordia University, 7141 Sherbrooke Street West, Montreal, QCH4B 1R6, Canada; Montreal Neurological Institute, McGill University, 3801 University Street, Montreal, QC H3A 2B4, Canada; International Laboratory for Brain, Music and Sound Research (BRAMS), 90 Vincent-d’Indy Avenue, Outremont, QC H2V 2S9, Canada; Centre for Research in Brain, Language and Music (CRBLM), 3640 de la Montagne Street, Montreal, QC H3G 2A8, Canada

**Keywords:** auditory expectancy, cortical–subcortical interactions, abstract rules, predictive coding, high-field fMRI

## Abstract

Perception integrates both sensory inputs and internal models of the environment. In the auditory domain, predictions play a critical role because of the temporal nature of sounds. However, the precise contribution of cortical and subcortical structures in these processes and their interaction remain unclear. It is also unclear whether these brain interactions are specific to abstract rules or if they also underlie the predictive coding of local features. We used high-field 7T functional magnetic resonance imaging to investigate interactions between cortical and subcortical areas during auditory predictive processing. Volunteers listened to tone sequences in an oddball paradigm where the predictability of the deviant was manipulated. Perturbations in periodicity were also introduced to test the specificity of the response. Results indicate that both cortical and subcortical auditory structures encode high-order predictive dynamics, with the effect of predictability being strongest in the auditory cortex. These predictive dynamics were best explained by modeling a top–down information flow, in contrast to unpredicted responses. No error signals were observed to deviations of periodicity, suggesting that these responses are specific to abstract rule violations. Our results support the idea that the high-order predictive dynamics observed in subcortical areas propagate from the auditory cortex.

## Introduction

Perceptual processes often involve an active component, in which predictions about the environment are used to process incoming sensory information (Friston 2005). In audition, this cognitive mechanism contributes to deviance detection—a neural response signaling unexpected or unusual sensory information as sounds unfold in time (Grimm and Escera 2012). Comparing internal prediction with external inputs generates an error signal, allowing adjustment of mental models accordingly. The hierarchical organization of the auditory system that underlies this mechanism has been described in animal models (Carbajal and Malmierca 2018), which show that subcortical structures display local (low-level) predictive dynamics, resulting in adaptation to repeated sounds and enhanced responses to novel ones, while cortical structures encode more abstract predictions pertaining to patterns of sounds extended over time (Parras et al. 2017). The latter conclusion is consistent with the observation that the auditory cortex (AC) suppresses responses to deviant stimuli as these become more predictable (Kok and de Lange 2015). More generally, substantial evidence suggests that higher-order prediction signals can be dissociated from low-level adaptation effects (Wacongne et al. 2011; Todorovic and de Lange 2012).

The precise roles of auditory cortical and subcortical structures in these predictive processes, and especially the interaction between them, remain unclear, however, especially in humans. Recent functional MRI data show that the subcortical nuclei, inferior colliculi (IC), and medial geniculate bodies (MGB) generate prediction error signals in response to deviant tones (Cacciaglia et al. 2015) and that these responses are modulated by the predictability of the patterns (Tabas et al. 2020). These findings may be interpreted as evidence that subcortical areas compute higher-level predictions intrinsically or instead, they may represent predictive signals originating in cortical areas that propagate to subcortical areas (Tabas and von Kriegstein 2021). Since prior studies have only examined cortical and subcortical responses independently, and not their interactions, the respective roles of these structures in predictive processes remain unknown.

The putative substrate for cortical–subcortical functional interactions is provided by anatomical studies that describe separate afferent (lemniscal) and efferent (nonlemniscal) pathways that interconnect IC, MGB, and AC (Carbajal and Malmierca 2018), allowing for bottom–up and top–down information flow, respectively, and are hence suggested to play a role in predictive mechanisms (Asilador and Llano 2020). To date, however, the hypothesis that subcortical brain signals are modulated by expectancy because of cortico-subcortical interactions has not been tested in humans. Furthermore, it is not clear whether these brain modulations are specific to predictions derived from abstract rules, such as occur in sequences, or if they also underlie the predictability of local features, such as periodicity. Recent evidence from time-resolved methods (i.e. magnetoencephalography and intracranial electroencephalography recordings) has suggested that periodicity related to encoding of pitch information involves a mechanism of oscillatory entrainment, likely localized to early auditory cortical regions or thalamocortical circuits (Coffey et al. 2021; Lerousseau et al. 2021). As an oscillatory circuit can be considered as a low-level form of temporal prediction (Hovsepyan et al. 2020), we therefore explore whether a perturbation to periodicity is observed in the hemodynamic response.

Here, we conducted a high-field functional magnetic resonance imaging (MRI) experiment to investigate cortico-subcortical interactions during auditory predictive coding at different levels of information processing. We hypothesized that: (i) higher-order predictive dynamics (such as those observed in predicting deviant tones in a sequence) are manifested in cortical as well as subcortical areas, as seen in previous studies (Kok and de Lange 2015; Tabas et al. 2020; Tabas and von Kriegsten 2024); (ii) these predictive dynamics originate at the cortical level and propagate to subcortical areas; and (iii) local perturbations of stimulus periodicity are reflected only in subcortical structures. We developed a paradigm similar to that used in Tabas et al. (2020), in which regular streams of sounds are presented followed by deviants associated with different levels of expectancy, acquiring both cortical and subcortical hemodynamic signals using 7T fMRI, which is sensitive to both (Berlot et al. 2020). We also introduced orthogonal perturbations in the periodicity of the stimuli to test the specificity of the phenomena under study. We tested the hypotheses by examining hemodynamic responses to deviance as modulated by predictability in each structure, and by modeling cortical–subcortical interactions using path analysis, which allows probabilistic directional analysis (McIntosh and Gonzalez-Lima 1994; Protzner and McIntosh 2006). We used a precision neuroscience approach (Poldrack, 2017), employing a multi-level modeling strategy to characterize individual distributions. This approach allowed us to study the phenomena of interest with sufficient statistical power in a limited sample (Chen et al. 2012), rather than averaging across brain volumes, which results in loss of precision.

## Materials and methods

### Participants

Ten healthy human adults (6 female) aged 19 to 23 (mean age 21.7, standard deviation = 1.83), participated in the study. They were asked to fill out a demographic questionnaire, a health questionnaire to confirm the absence of neurological and audiological conditions, and the Montreal Music History Questionnaire (MMHQ) to document their musical training experience (Coffey et al. 2011). Subjects gave written informed consent and were compensated for their time. The experimental protocol was approved by the Research Ethics Board at McGill University.

### Experimental paradigm

#### Oddball task

To study the neural response to stimuli as a function of predictability, we used an oddball task where the expectancy of the different stimulus was experimentally manipulated, adapted from Tabas et al. (2020). The oddball paradigm has successfully been used in fMRI settings to observe both adaptation and deviant detection from blood-oxygen-level-dependent (BOLD) signals (Horovitz et al. 2002; Liebenthal et al. 2003). It consisted of four runs, each containing 6 blocks of 9 trials. The intertrial interval (ITI) was jittered with a silent gap between 1.5 and 11 s and an average of 5 s to keep subjects from predicting when the next sequence would begin, as well as to maximize the estimation of the neural responses. In each trial, participants listened to a sequence of 8 tones with 700 ms inter-stimulus intervals (ISIs). Among these, seven tones were repetitions of the same stimulus (henceforth standard stimuli), while the remaining tone was different in pitch (henceforth deviant stimulus).

The tones used in the experiment consisted of 250 ms synthesized complex waves with fundamental frequencies as follows (corresponding Western musical scale value given in parentheses): 73 (D2), 87 (F2), and 98 Hz (G2). Each tone comprised 4 harmonics, with successive harmonics −3 dB with respect to the one before. To create the stimuli for the low-level feature manipulation (see below), two additional versions of each tone were created in which 15 ms of the tone starting at 150 ms poststimulus onset was replaced by a pink noise burst (scaled to be 25% of the amplitude of the tone). In one of these versions, the phase of the tone after the pink noise continued as if there had been no interruption, and in the other one, the phase was inverted (i.e. multiplied by −1). Five-millisecond raised cosine ramps were applied at the beginning and end of each stimulus segment (i.e. tone, noise) to avoid clicks.

**Fig. 1:**
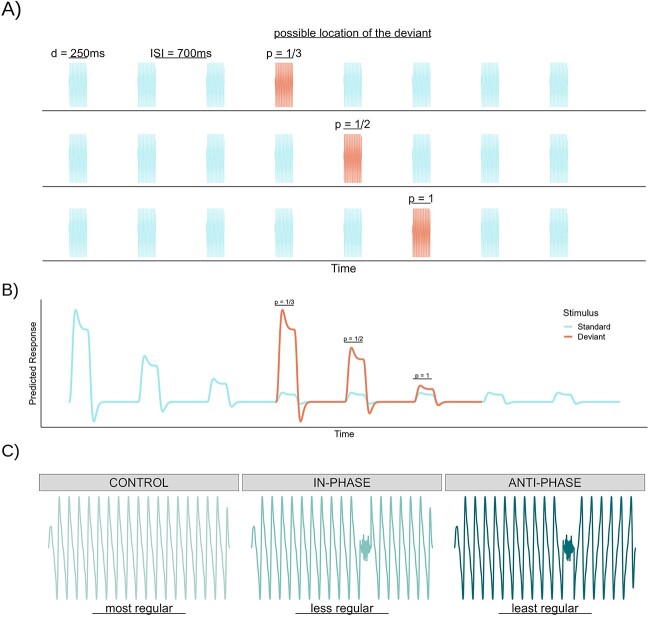
Experimental design. A) Oddball task (Tabas et al. 2020). Participants listened to sequences of eight tones where one tone was different in pitch, which they had to identify. This deviant stimulus could take place in three different positions with different levels of predictability assigned (from top to bottom): fourth (*P* = 1/3, least certain), fifth (*P* = 1/2, more certain), or sixth (*P* = 1, most certain). B) Predicted neural responses to the stimuli making a sequence. Greater responses are expected for the first tone in the sequence, as well as for the least predicted deviant. Conversely, habituation is expected to follow repeated standard tones, and a suppression of the response to the deviant is expected as a function of expectancy. C) Low-level feature manipulation. Orthogonally, blocks were randomly assigned to one of three conditions: a control condition with no perturbation (most regular); a condition with a short noise perturbation (less regular); and a condition returning in antiphase after the same perturbation (least regular).

Each run was segmented into six randomly presented blocks, where each block consisted of a unique paired combination of these tones to serve as the standard and deviant. To study different levels of expectancy assigned to the oddball, the deviant stimulus always appeared in either the fourth, fifth, or sixth position of the stream, generating three different types of sequence (Fig. 1A) as in Tabas et al. (2020). Since, however, the listener has no way to know a priori whether the deviance will occur in position 4, 5, or 6, the expected probability of occurrence right before the deviant is different for each position. Thus, the probability that the deviant will happen in position 4 after 3 repetitions of the standard is one-third (*P* = ⅓, least certain). If the deviant has yet not occurred in position 4, then the listener knows that the probability of occurrence for position 5 is ½ because there are only two possible outcomes left (*P* = ½, more certain). Finally, if the deviant has not occurred yet in positions 4 or 5, then it must occur on position 6 with complete certainty (*P* = 1, most certain). The deviant stimulus in each one of these sequences was expected to elicit different levels of activity as a function of their certainty of occurrence, with weaker responses associated with greater predictability (Fig. 1B; Tabas et al. 2020). After each sequence was completed, participants were asked to indicate the position of the deviant as accurately and quickly as possible via a button press using their left hand in order to ensure that they correctly identified the position of the stimuli. The order of blocks, trials, and position of the deviants was randomized, but with the constraint that each deviant position should take place exactly 18 times per run. Therefore, while participants knew the possible locations of the deviant stimulus, they could not predict its exact position until they listened to the sequences. In addition, 21 silent trials of the same duration as the tone sequences were randomly introduced in each run to establish a stable baseline. Each run lasted around 13 min, depending on the ITI jitters. Runs were separated by breaks of 1 min for the participants to rest. The first run was preceded in the scanner by a practice run of four tone sequences and one silent trial to ensure that subjects understood the task and could hear the stimuli clearly over the scanner noise.

Stimuli were presented using PsychoPy (Peirce et al. 2019) and delivered binaurally through Sensimetrics S15 earphones. Loudness was adjusted independently for each subject to a comfortable level before starting the data acquisition (LAeq range = 67 to 72 dB sound pressure level [SPL]).

#### Phase flip manipulation

To investigate whether similar dynamics are also observed at the level of local acoustic features, we introduced an experimental manipulation that was orthogonal to the oddball task. For this purpose, the blocks in each run were assigned to three different conditions where the periodicity of the stimuli was manipulated to different extents. The three conditions consisted in: tones with no perturbation (control condition, most regular); tones with a small noise perturbation, continuing thereafter (in-phase condition, less regular); and tones with the same perturbation, continuing in anti-phase after the noise (anti-phase, least regular) (Fig. 1C). The difference in phase polarity after the perturbation was not perceivable according to the results of a separate pilot experiment (*n* = 10 participants, *k* = 600 trials, in a same–different task; performance was never above chance). The noise perturbation consisted of an approximately one-cycle-long pink noise excerpt (15 ms) taking place at about two-thirds of the stimulus length (150 ms). Each block contained tones that had been manipulated in the same way, and blocks were randomly and evenly assigned to each one of these conditions.

### Data acquisition

MRI data were acquired with the Siemens Magnetom 7 Tesla scanner installed at the McConnell Brain Imaging Centre of the Montreal Neurological Institute and Hospital, using a 32-channel head coil.

**Fig. 2:**
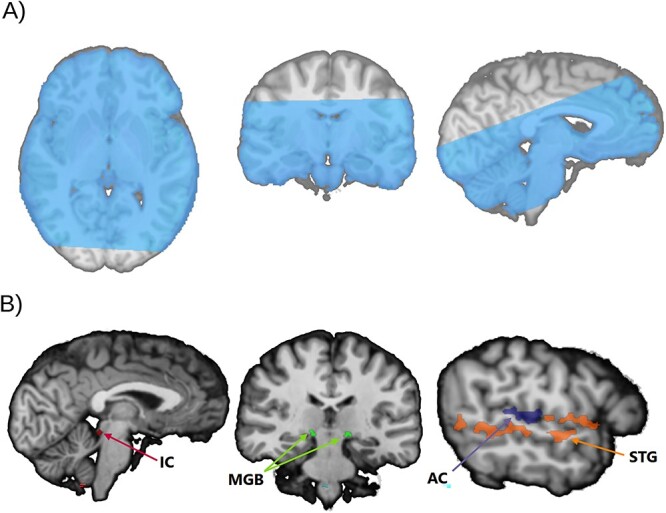
A) Partial field of view for fMRI acquisition encompassing all relevant ROIs. B) Bilateral ROIs reverse-normalized into native space. IC: inferior colliculus; MGB: medial geniculate body; AC: auditory cortex; STG: superior temporal gyrus.

We used a field of view (FoV) of 225 mm × 225 mm yielding partial brain coverage with 48 slices (7.2 cm). This volume was oriented in parallel to the superior temporal gyrus such that the slices encompass the IC, MGB, and superior temporal gyrus (Fig. 2A). In addition, we acquired 15 resting whole-brain EPI volumes with the same parameters (including the FoV) and 86 slices that were used to help align the slab of slices acquired during task fMRI with the whole-brain anatomical image. Field maps using gradient echo were also acquired for the two types of sequence to estimate the geometric distortions caused by the field inhomogeneities.

Functional MRI data during task performance were acquired using echo planar imaging (EPI) sequences. The EPI sequence was acquired with the following parameters: TR = 1,530 ms, TE = 25.4 ms, flip angle = 67°, GRAPPA with acceleration factor 2, phase partial Fourier = 7/8, voxel size = 1.5 mm isotropic, interleaved acquisition, and anterior to posterior phase-encode direction (Griswold et al. 2002). Structural images were recorded using an MP2RAGE T1 protocol with 700 μm isotropic resolution, TE = 2.33 ms, TR = 5,000 ms, TI1 = 900 ms, TI2 = 2,750 ms, flip angle 1 = 4°, flip angle 2 = 3°, FoV = 240 mm × 240 mm, GRAPPA acceleration factor 3 (Marques et al. 2010).

### Data preprocessing

The preprocessing pipeline was batched in SPM12 (Penny et al. 2011). First, slice-timing correction was applied to the first slice. Next, SPM’s FieldMap Toolbox was used to estimate the geometric distortions in both the partial- and full-brain functional images. We then used SPM’s *Realign and Unwarp* tool to perform motion and distortion correction on the partial- and full-brain functional data. Coregistration of the anatomic image was performed using SPM’s *Coregister* tool in two sequential steps: the mean full-brain image was aligned with the mean partial image, followed by the coregistration of the anatomic image to the mean full-brain image. The anatomic image underwent a segmentation process. The images were then smoothed using a 2 mm full width at half maximum (FWHM) Gaussian kernel.

### Region of interest definition

Cortical regions of interest (ROIs) were defined bilaterally with Brodmann areas corresponding to primary and adjacent regions of the auditory cortex (AC: areas 41 and 42), and to auditory regions outside the peri-primary areas (STG: area 22) using Lancaster et al. (2000) Talairach atlas. The IC and MGB were defined bilaterally using an auditory-dedicated atlas for subcortical structures (Sitek et al. 2019). The choice of bilateral ROIs was motivated by previous literature that did not report hemispheric differences of the effects under study at either the cortical or subcortical level (Tabas et al. 2020; Tabas and von Kriegsten 2024) and to maximize signal-to-noise ratios. The ROIs, initially defined in montreal neuroligical institute (MNI) space, were reverse-normalized into each participant’s native space, using the inverse deformation field obtained from the segmentation of the anatomic image (Fig. 2B).

## Statistical analysis

(For a full mathematical view of the models employed in this study, please see the Supplementary Materials.)

### Oddball task

#### Behavioral data

To test whether participants could correctly identify the position of the deviant stimulus, a 3-alternative forced choice (3AFC), multilevel signal detection theory model (DeCarlo 2012) was fit to the data using Bayesian inference. This model estimates the observers’ sensitivity (d’) in the perception of the target stimulus at both the group and individual levels.

#### Brain data

##### First-level analysis

To estimate the mean neural response to each type of stimulus in the oddball task, a general linear model (GLM) was fit to every individual and ROI’s data course using SPM and the MarsBar toolbox (Brett et al. 2002; Penny et al. 2011) as an event-related design. This method has been used successfully to model auditory adaptation and deviant detection in studies with subsecond ISIs (e.g. Lumaca et al. 2019). These models included conditions for the first standard appearing in each sequence (std0), the standards before the deviant (std1), each type of deviant (position 4: dev4, position 5: dev5, position 6: dev6), and the standards after the deviant (std2). Parametric modulations of position were also included for std1 and std2 in order to control for sequential effects in these categories, and the movement parameters from realignment were included as regressors of no interest. The estimated means of each condition were extracted together with their associated standard errors (SEs) for further analysis.

##### Second-level analysis

To study differences between brain responses to each type of stimulus at the group level, a mixed-effects multilevel model was fit to the first-level mean estimates with their associated SEs using Bayesian inference. This allowed us to pool the first-level variances as opposed to simply aggregating the mean estimates, thus preserving the first-level analysis precision and maximizing statistical power (Chen et al. 2012; also see Vicente et al. 2023 for recent work using this approach). The outcome of the model consisted in a multivariate response with all ROI neural responses estimated as correlated. Random effects of participant were included. We focused on four comparisons of interest to our research questions: standard adaption (std0-std1), deviant detection (dev4-std1), predictive suppression (dev4-dev6), and complete suppression (dev6-std2). Given that data were modeled in a multivariate fashion (i.e. with activity in each ROI being the dependent variables), the correlations between activity in the different ROIs returned by the model were explored.

##### Path analysis of initial tone detection

To study the most likely directionality of initial tone detection along the auditory pathway (i.e. top–down vs. bottom–up processing), we compared two path analyses with opposing directions. Path analysis is a probabilistic method that assesses the influence that different nodes have on each other and has been proposed to study directional connectivity in cognitive neuroscience (McIntosh and Gonzalez-Lima 1994; Protzner and McIntosh 2006). Importantly, this method allows the direct comparison of competing models by assessing each model’s fit to the predicted data. Therefore, if activity in area A is better predicted from activity in area B than from activity in area C, model comparison will favor the former path over the latter.

For this purpose, we fitted the first-level neural responses to the initial tone in the sequences (i.e. std0) with measurement error using Bayesian inference. Similar to the second-level analysis, first-level variances were also considered, both for dependent variables and for independent variables (McElreath 2018). In the top–down model, STG activity was estimated from its average, AC activity was predicted from STG activity, MGB activity was predicted from AC activity, and IC activity was estimated from IC activity. On the contrary, in the bottom–up model, IC activity was estimated from its average, MGB activity was predicted from IC activity, AC activity was predicted from MGB activity, and STG activity was predicted from AC activity. Finally, an intercept-only model was also implemented, where the activity in each ROI was only estimated from their own average. This was done to ensure that the set of paths in the top–down and bottom–up models predicted the data better than a null model containing no directional paths. The three models were compared using the widely applicable information criterion (WAIC) for Bayesian models (Watanabe, 2010). The WAIC is an index that assesses model fit, with lower values indicating a better fit to the predicted data. Moreover, the difference in model fit between competing models comes with an associated SE. If the associated SE is smaller than the absolute difference, the result is interpreted to be significant.

##### Path analysis of predictive suppression

Similarly, in order to study the most likely directionality of the predictive suppression effect, we compared two path analyses with opposing directions plus an intercept-only model. In these models, we only included deviant observations from areas showing predictive and/or complete suppression of the deviant in the second-level analysis. Also for this case, and to avoid the possibility that differences in absolute amplitude might explain the results, we standardized the first-level data per subject and ROI. For the bottom–up and top–down models, deviant position predicted the first ROI in the path. Random effects of participant were included in these models.

### Phase flip manipulation

#### Brain data

##### First-level analysis

To estimate the mean neural response to each type of stimulus of the low-level features manipulation, a general linear model (GLM) was fit to every individual and ROI’s data course using SPM and MarsBar. This model included conditions for stimuli with no embedded pink noise, stimuli with embedded pink noise returning in-phase, and stimuli with embedded pink noise returning in anti-phase. The movement parameters from realignment were included as regressors of no interest. These estimated means were extracted together with their SEs for further analysis.

##### Second-level analysis

To study differences between brain responses to each type of stimulus at the group level, a mixed-effects multilevel model was fit to the first-level estimates with their associated SEs using Bayesian inference. The outcome of the model consisted in a multivariate response with all ROI neural responses estimated as correlated. Random effects of participant were included.

### Model estimation and null-hypothesis testing

Posterior distributions were approximated with 6 Markov chains of 2,000 samples with no thinning, burning-in the first 1,000 samples. These parameters were chosen to make sure that sufficient samples were drawn to characterize posterior distributions reliably. Samples were drawn with the no-U-turn algorithm (Stan development team 2022) through the R interface brms (Bürkner 2017) in R (R core team 2021). All chains were initialized at zero. All model parameters converged as indicated by Gelman’s split-R-hat equaling 1 (Gelman et al. 2013).

In order to perform null-hypothesis testing, we computed the probability of direction (PD) of the posterior distributions (an index representing the certainty that an effect goes in a particular direction, be it positive or negative) and converted this index to a two-sided *P*-value through the formula *2(1—PD)* (Makowski et al. 2019). We used a threshold of *P* < 0.05 to decide on the certainty of the effects. The reported centrality of the estimates is the maximum a posteriori (MAP) probability estimate (i.e. the peak of the posterior distribution; Kruschke 2015). We also report Bayes factors of evidence for the alternative hypothesis over the null hypothesis (BF_10_) and posterior 95% highest density intervals (HDI_95_) for completeness (Kruschke 2015), together with trends represented as β values and differences represented as Δ values.

Note that multiple comparisons adjustment is not necessary under this inference framework. In brief, approximating the likelihood of the parameters given the data does not incur in the type-I error rate inflation that characterizes multiple frequentist testing (Han and Park 2018). Moreover, the regularization toward the null hypothesis that the hierarchical structure of the effects and the prior distributions impose on the parameters make Bayesian testing analytically more conservative (Kruschke 2015).

## Results

### Oddball task

#### Behavioral data

**Fig. 3:**
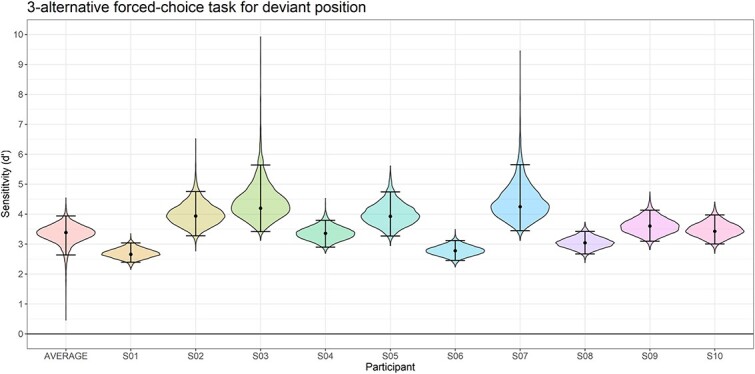
Sensitivity (*d’*) for the oddball position. Participants displayed significant above-zero sensitivity in identifying the position of the deviant stimulus in the sequences. Violin plots represent the posterior distributions. Effects are summarized with maximum a posteriori estimates and their associated 95% highest density intervals.

Participants showed high sensitivity at both the group (d’ = 3.38, HDI_95_ = 2.71 to 3.94, BF_10_ = 8,970, *P* < 0.001) and all individual levels (Fig. 3), meaning that they correctly identified the position of the deviant stimulus throughout the experiment.

#### Brain data

##### Second-level analysis

**Fig. 4:**
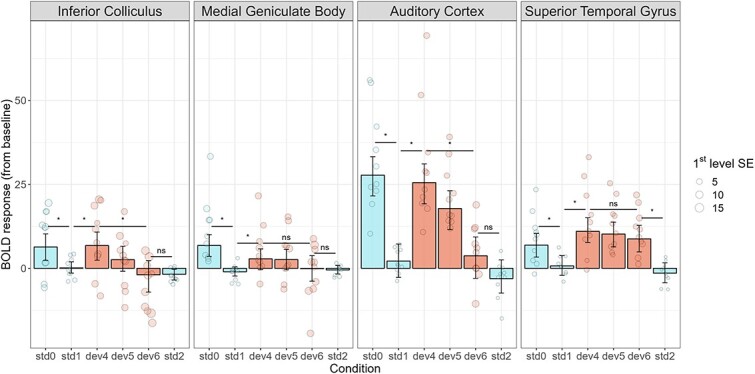
Neural responses to each type of stimulus in the oddball task, for each ROI. Significant adaptation (std1-std0) and deviant detection (dev4-std0) was observed in all four ROIs. Predictive suppression (dev4-dev6) was significant in the IC and the AC, but not in the MGB or STG. Complete suppression (dev6-std2) took place in the IC, MGB, and AC, but not in the STG. std0: first tone in the sequence; std1: repeated tones before the deviant; dev4, dev5, and dev6: deviant tone in positions 4, 5, and 6, respectively; std2: repeated tones after the deviant. IC: inferior colliculus; MGB: medial geniculate body; AC: auditory cortex; STG: superior temporal gyrus. Group effects are summarized with maximum a posteriori estimates and their associated 95% highest density intervals. First-level standard errors are represented with different point sizes.

**Fig. 5:**
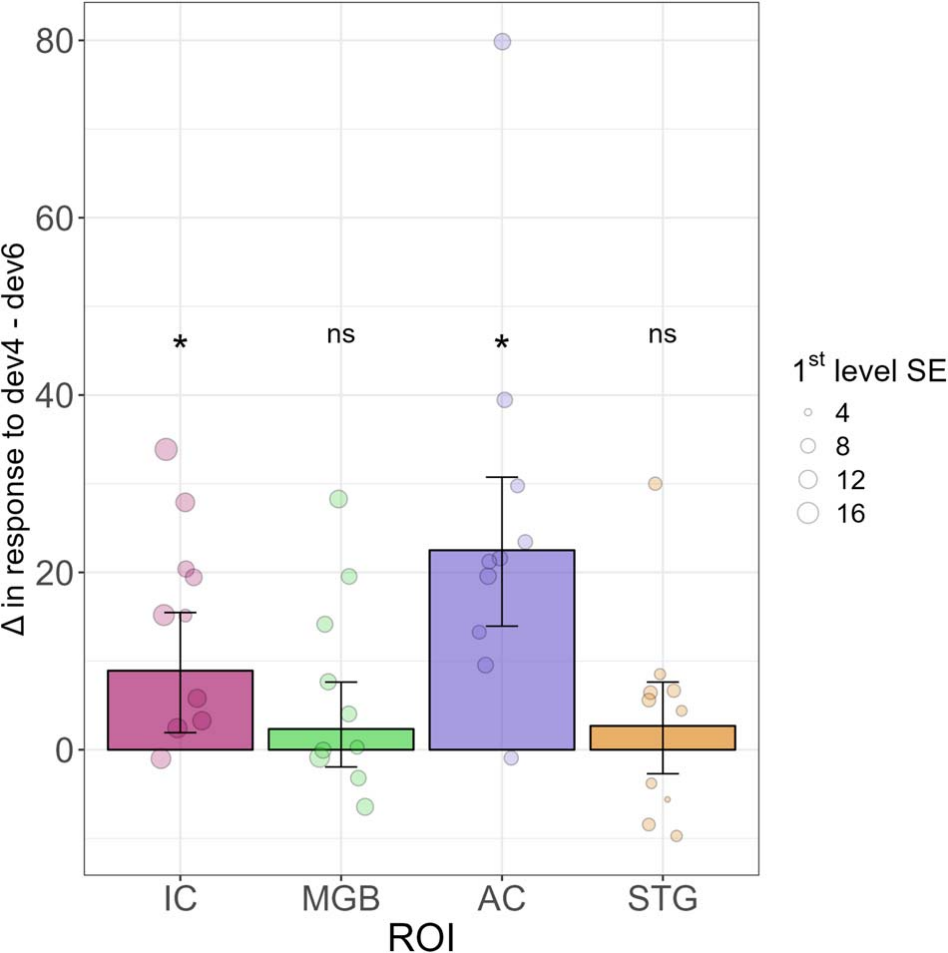
Magnitude of the predictive suppression effect (dev4-dev6) in the four ROIs. The effect was significant in the IC and AC, but not in the MGB or STG. dev4 and dev6: deviant tone in positions 4 and 6, respectively. IC: inferior colliculus; MGB: medial geniculate body; AC: auditory cortex; STG: superior temporal gyrus. Group effects are summarized with maximum a posteriori estimates and their associated 95% highest density intervals. First-level standard errors are represented with different point sizes.

We observed positive differences between std0 and std1 in all four ROIs (IC: Δ = 6.23, HDI_95_ = 1.68 to 10.52, BF_10_ = 43.4, *P* < 0.01; MGB: Δ = 7.68, HDI_95_ = 4.04 to 11.58, BF_10_ = 729, *P* < 0.001; AC: Δ = 25.72, HDI_95_ = 17.75 to 33, BF_10_ = 217,000, *P* < 0.001; STG: Δ = 5.92, HDI_95_ = 1.4 to 10.59, BF_10_ = 35.88, *P* < 0.05), meaning adaptation took place as the stimuli repeated at both the cortical and subcortical levels. We also observed positive differences between dev4 and std1 in all four ROIs (IC: Δ = 6.63, HDI_95_ = 1.54 to 10.88, BF_10_ = 53.58, *P* < 0.05; MGB: Δ = 3.66, HDI_95_ = 0.18 to 7, BF_10_ = 13.32, *P* < 0.05; AC: Δ = 23.3, HDI_95_ = 15.37 to 30.96, BF_10_ = 97,900, *P* < 0.001; STG: Δ = 10.08, HDI_95_ = 5.63 to 14.94, BF_10_ = 1960, *P* < 0.001), meaning deviant detection took place when a deviant occurred under uncertainty at both the cortical and subcortical levels. We observed positive differences between dev4 and dev6 in the IC and the AC (IC: Δ = 9.4, HDI_95_ = 1.98 to 15.76, BF_10_ = 40.12, *P* < 0.01; AC: Δ = 22.9, HDI_95_ = 13.70 to 30.2, BF_10_ = 59,300, *P* < 0.001), meaning that predictive suppression took place as the deviant became more predictable at both the cortical and subcortical levels. However, we did not observe this effect in the MGB or the STG, although the trend was also positive (MGB: Δ = 2.6, HDI_95_ = −1.8 to 7.81, BF_10_ = 3.07, *P* = 0.26; STG: Δ = 1.89, HDI_95_ = −2.78 to 7.43, BF_10_ = 2.69, *P* = 0.41) (Fig. 5). Finally, we did not observe differences between dev6 and std2 in the IC, MGB, and AC (IC: Δ = −0.33, HDI_95_ = −5.39 to 4.22, BF_10_ = 1.72, *P* = 0.84; MGB: Δ = 0.54, HDI_95_ = −3.52 to 4.14, BF_10_ = 1.41, *P* = 0.85; AC: Δ = 5.34, HDI_95_ = −2.37 to 13.38, BF_10_ = 8.16, *P* = 0.16). However, we did observe a positive difference in the STG (STG: Δ = 10.05, HDI_95_ = 5.16 to 15.02, BF_10_ = 1,690, *P* < 0.001), suggesting that, unlike the other regions, this area might be sensitive to all deviants regardless of position (Fig. 4).

**Fig. 6:**
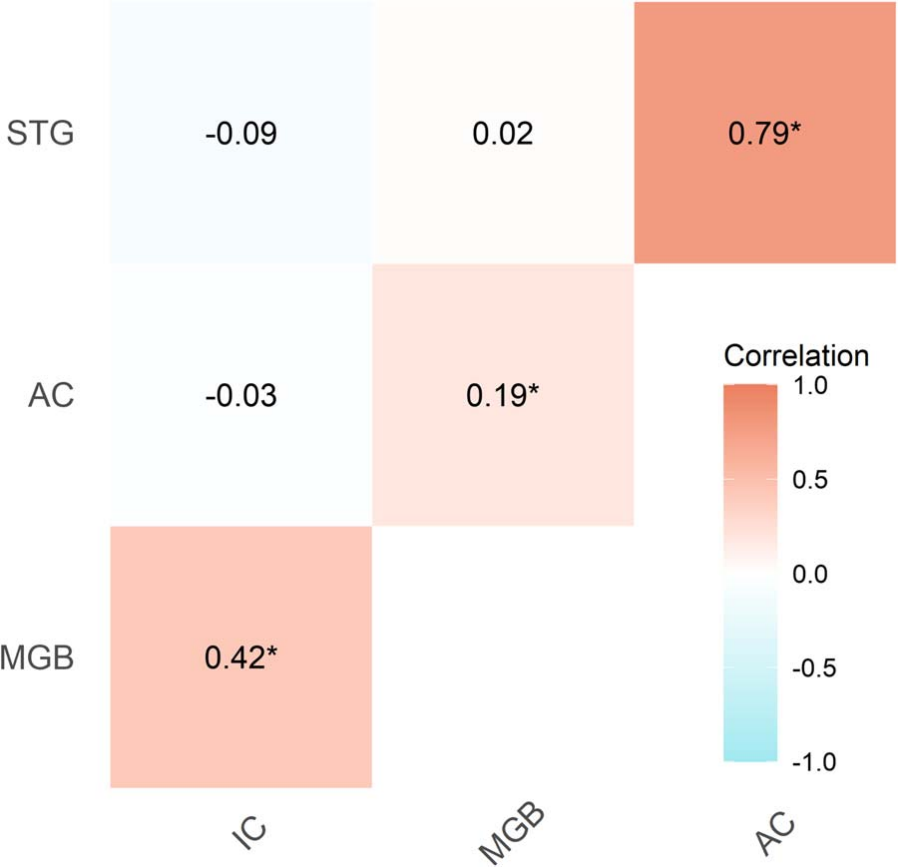
Correlation matrix between ROI activity in the oddball task. Adjacent areas of the pathway are positively correlated, but not non-adjacent areas. IC: inferior colliculus; MGB: medial geniculate body; AC: auditory cortex; STG: superior temporal gyrus. Correlation scores are summarized with maximum a posteriori estimates.

Finally, the correlations between the different dependent variables in the model (i.e. activity in the different ROIs) were positive between adjacent areas of the pathway (IC↔MGB: *r* = 0.42, HDI_95_ = 0.22 to 0.56, BF_10_ = 167.55, *P* < 0.001; MGB↔AC: r = 0.19, HDI_95_ = 0.02 to 0.37, BF_10_ = 2.46, *P* < 0.05; AC↔STG: *r* = 0.79, HDI_95_ = 0.68 to 0.88, BF_10_ = 7.82 × 10^20^, *P* < 0.001), but not significant otherwise (IC↔A1: *r* = −0.02, HDI_95_ = −0.23 to 0.18, BF_10_ = 0.29, *P* < 0.001; IC↔STG: *r* = −0.05, HDI_95_ = −0.27 to 0.12, BF_10_ = 0.38, *P* < 0.05; MGB↔STG: *r* = −0.004, HDI_95_ = −0.18 to 0.2, BF_10_ = 0.27, *P* < 0.001), displaying an anatomically coherent correlation structure of the data (Fig. 6).

##### Path analysis of initial tone detection

**Fig. 7:**
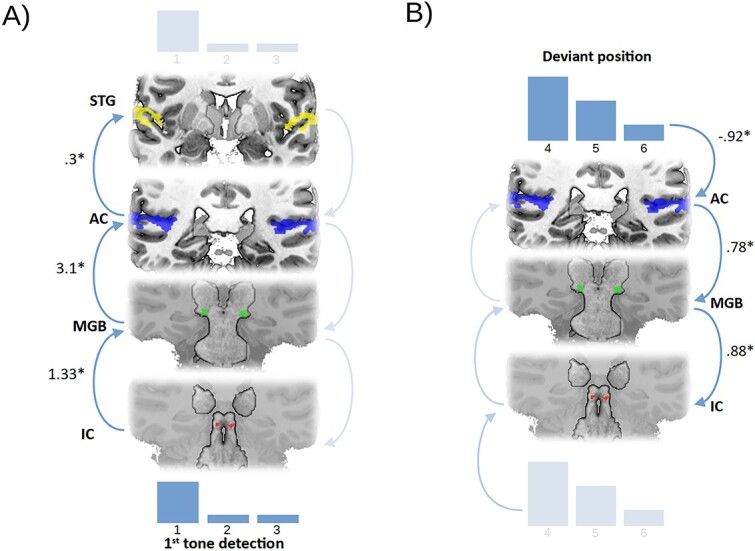
A) Effective connectivity analysis of first tone detection in the oddball task on all ROIs. The bottom–up model explains the data better than its top–down counterpart, with activity in subcortical areas predicting activity in cortical areas. B) Effective connectivity analysis of the deviant position effect in the oddball task on ROIs showing predictive suppression. The top–down model explains the data better than its bottom–up counterpart, with deviant position predicting less cortical activity, which, in turn, predicts activity in subcortical areas. IC: inferior colliculus; MGB: medial geniculate body; AC: auditory cortex; STG: superior temporal gyrus. Beta weights are summarized with maximum a posteriori estimates.

In line with our hypothesis, the bottom–up path analysis modeling the response to the first (unpredicted) tone in the sequence explained the data better than its top–down counterpart (bottom–up_WAIC_ = 280.5, top–down_WAIC_ = 301.9, Δ_WAIC_ = −21.4, SE = 15.2). The winning model also outperformed the intercept-only model (intercept-only_WAIC_ = 360.7; Δ_WAIC_ = 79.8, SE = 11.2). As expected, all regression coefficients in this model were significant, with a positive effect of all paths in the model (IC → MGB: β = 1.33, HDI_95_ = 0.78 to 2.12, BF_10_ = 33,400, *P* < 0.001; MGB → AC: β = 3.1, HDI_95_ = 2.44 to 3.86, BF_10_ = 2.29 × 10^12^, *P* < 0.001; AC → STG: β = 0.3, HDI_95_ = 0.22 to 0.41, BF_10_ = 13,500, *P* < 0.001). Therefore, our results are consistent with the idea that neural signals associated with detection are generated in subcortical areas and propagated up to cortical areas of the auditory pathway (Fig. 7A).

##### Path analysis of predictive suppression

In line with our hypothesis, the top–down path analysis modeling the suppression of deviants as they became more predictable explained the data better than its bottom–up counterpart (bottom–up_WAIC_ = 270.9, top–down_WAIC_ = 246.8; Δ_WAIC_ = 24, SE = 5). The winning model also outperformed the intercept-only model (intercept-only_WAIC_ = 319.3; Δ_WAIC_ = 72.6, SE = 11.8). As expected, all regression coefficients in this model were significant, with a negative effect of deviant position predicting AC activity [position → AC: β = −0.92, HDI_95_ = −1.11 − (−0.77), BF_10_ = 4.75 × 10^9^, *P* < 0.001], and a positive effect for all subsequent paths in the model (AC → MGB: β = 0.78, HDI_95_ = 0.12 to 1.4, BF_10_ = 5.9, *P* < 0.05; MGB → IC: β = 0.88, HDI_95_ = 0.23 to 1.87, BF_10_ = 14.4, *P* < 0.05). Therefore, predictive suppression signals are most likely generated in cortical areas and propagated down to subcortical areas of the auditory pathway (Fig. 7B).

### Phase flip manipulation

#### Brain data

##### Second-level analysis

**Fig. 8:**
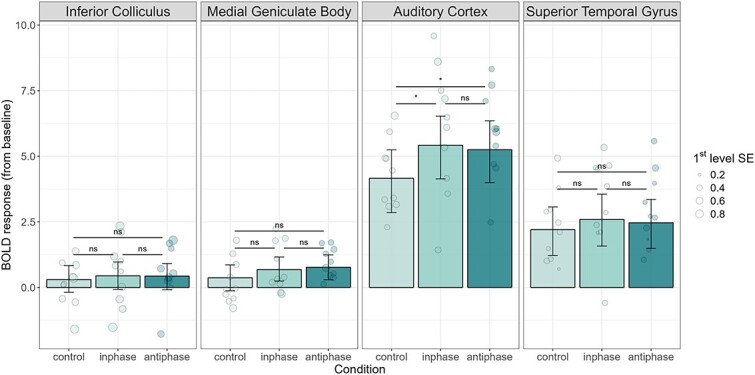
Neural responses to each type of stimulus in the low-level features experimental manipulation, per ROI. No differences were observed between tones periodicity returning in phase and antiphase after the embedded pink noise in these conditions in any ROI. A difference in the magnitude of the response as only observed in the AC ROI between conditions with and without the embedded pink noise. Control: tones with no embedded pink noise; inphase: tones with embedded pink noise, returning in phase; antiphase: tones with embedded pink noise, returning in antiphase. IC: inferior colliculus; MGB: medial geniculate body; AC: auditory cortex; STG: superior temporal gyrus. Group effects are summarized with maximum a posteriori estimates and their associated 95% highest density intervals.

We did not observe statistically significant differences in any ROI between the conditions in which sounds returned in-phase after the pink noise disruption as compared to the condition in which sounds returned in anti-phase after the same disruption (Fig. 8; IC: Δ = −0.0006, HDI_95_ = −0.55 to 0.4, BF_10_ = 0.057, *P* = 0.82; MGB: Δ = 0.002, HDI_95_ = −0.41 to 0.62, BF_10_ = 0.12, *P* = 0.74; AC: Δ = 0.07, HDI_95_ = −1.03 to 0.71, BF_10_ = 0.29, *P* = 0.85; STG: Δ = −0.003, HDI_95_ = −0.91 to 0.47, BF_10_ = 0.11, *P* = 0.61). The only notable difference was between the conditions with the embedded pink noise and the condition without it in the AC (inphase-control: Δ = 1.12, HDI_95_ = 0.30 to 2.28, BF_10_ = 13.53, *P* < 0.01; antiphase-control: Δ = 1.1, HDI_95_ = 0.23 to 2.11, BF_10_ = 7.01, *P* < 0.05), but not in the IC (inphase-control: Δ = 0.002, HDI_95_ = −0.34 to 0.72, BF_10_ = 0.076, *P* = 0.61; antiphase-control: Δ = X, HDI_95_ = −0.4 to 0.57, BF_10_ = 0.07, *P* = 0.75), MGB (inphase-control: Δ = 0.01, HDI_95_ = −0.23 to 0.89, BF_10_ = 0.20, *P* = 0.28; antiphase-control: Δ = 1.1, HDI_95_ = −0.09 to 1.06, BF_10_ = 0.19, *P* = 0.18) or STG (inphase-control: Δ = 0.01, HDI_95_ = −0.23 to 1.27, BF_10_ = 0.17, *P* = 0.28; antiphase-control: Δ = 0.005, HDI_95_ = −0.39 to 1.01, BF_10_ = 0.13, *P* = 0.46). Therefore, although the presence of noise generated a neural signal, there was no evidence of sensitivity to phase-related modulations of the periodicity in the BOLD signal.

## Discussion

The present study clarifies the role of cortical and subcortical deviance detection mechanisms in the auditory system. Prior fMRI studies using a similar protocol reported that both AC and subcortical auditory nuclei (IC and MGB) demonstrate deviance detection (Cacciaglia et al. 2015), and its suppression as a function of predictability in tone sequences (Tabas et al. 2020; Tabas and von Kriegsten 2024). We confirm these findings, but based on our effective connectivity analysis of cortical-to-subcortical influences, we conclude that the subcortical responses represent propagation of predictive signals from higher-order regions to subcortical nuclei, rather than being intrinsic responses within the subcortical structures. In addition, we show that these responses are specific to predictions of stimuli within patterns, as opposed to predictions of local features such as periodicity.

Several aspects of our design help to validate the conclusions from our study. First, we present behavioral data indicating that all volunteers were able to perform the task at high levels of accuracy (Fig. 3). Second, the ROIs for both cortical and subcortical regions were defined independently of the functional data using validated anatomical atlases (Lancaster et al. 2000; Sitek et al. 2019), thus avoiding any bias. Third, the pattern of intercorrelation of activity across the four ROIs yielded significant correlations only for adjacent structures that are most anatomically linked within the auditory pathway (IC–MGB, MGB–AC, and AC–STG). Although anatomical connections between some nonadjacent structures do exist, they are much less prominent than the direct ones (Powell and Hatton 1969; Terreros and Delano 2015; Williamson and Polley, 2019). This adjacent-only correlation structure has also been observed in resting-state studies of the auditory pathway (Chandra et al. 2024). Therefore, the pattern we observed follows as expected from the hierarchical organization of this system (Fig. 6) and provides further validation for the measures obtained. Finally, we observed significant adaptation in the BOLD signal in all four ROIs when comparing the second standard tone to the first one, indicating significant sensitivity to repetition with the present experimental design and analysis strategy. We used a precision neuroscience approach (Poldrack, 2017) which allows the statistical modeling of each individual and which is more powerful than the intersubject averaging approach commonly used in neuroimaging (Chen et al. 2012); it also allows us to take advantage of the high anatomical resolution afforded by 7T fMRI, as minimal smoothing was applied to the functional data.

The operation of the prediction mechanism is shown by the attenuation of the response as a function of predictability in the IC and AC that cannot be explained by habituation only: If this latter effect were true, the appearance of a deviant stimulus would elicit new neural responses regardless of its predictability (i.e. the response would be the same for all deviant positions within the pattern). On the contrary, however, we observe the strongest activity when the deviant stimulus is most unexpected (in the first of three possible positions), with a progressive inhibition as it becomes more expected and little to no response when it’s most expected (in the third position)—much like the lack of response to the repeated standard stimuli after the first occurrence. The evidence for prediction in the MGB is more limited, likely related to the relatively low signal magnitude within this region. However, within this structure, there seemed to be complete suppression of the fully predicted sound (dev6), because the magnitude of response was no different from the following standard stimulus (std2). This suppression was also observed for the IC and AC, suggesting that for all three structures, once the deviant tone had not occurred in positions 4 or 5, there was no uncertainty about its appearance in position 6, leading to a complete suppression, in line with a predictive coding model.

This predictive coding phenomenon has been demonstrated in prior studies. Our main contribution to this line of research is that we studied the interactions between subcortical and cortical areas within the auditory pathway using this experimental design. As expected, the predictive dynamics displayed by subcortical activity are also observed in the AC. To directly test the hypothesis of top–down inhibition, we carried out an analysis of effective connectivity in which we modeled the influence of each region in the hierarchy on the adjacent region to determine directionality of the effect. Notably, when modeling the response to the deviants, we found that the top−down model outperformed the bottom–up one in its predictions, suggesting that subcortical predictive responses can be explained by processing occurring at hierarchically higher levels. This conclusion is consistent with the predictive coding hypothesis of perception, where lower-level areas of the hierarchy are informed of predictions, and prediction errors readjust inferences with incoming information (Kok and de Lange 2015). This pattern, however, contrasted with the effective connectivity of initial tone detection, which was better explained by bottom–up information flow. Thus, the information flow for the first tone in the sequence, for which there is no obvious predictor, proceeds from subcortical to cortical structures. The dissociation between the two types of connectivity fits well with the idea that the hierarchy uses available information to efficiently encode stimuli, assigning relevance to novel stimuli (Grimm and Escera 2012). Thus, by studying the interaction between different regions of the auditory pathway, as opposed to studying each region separately, we could adduce evidence that pattern-related error signals are generated cortically and propagated to subcortical areas, as opposed to the view that they are generated locally in every step of the pathway.

At the cortical level, however, not all areas under study displayed the same dynamics. While AC activity fell in line with predictive coding principles, the STG did not show predictive suppression with predictability, nor did it show any evidence of suppression when comparing the fully predicted deviant (dev6) with the immediately following standard tone. Instead, the STG responded to all deviants regardless of how predictable they were, only showing adaptation to repeated stimuli. This finding suggests hierarchically distinct roles in the representation of auditory information carried out by different cortical areas. While early auditory areas might assign relevance in the encoding of novel stimuli following a predictive coding strategy, more downstream cortical areas might be involved in more abstract pattern representation, for which habituation/detection dynamics might be a better strategy. This interpretation would be in line with previous literature pointing out that whereas early areas of the hierarchy are sensitive to the magnitude of acoustic changes, more distal areas encode the more abstract aspect that a change has occurred, with less sensitivity to the specifics of what has changed (Schönwiesner et al. 2007). Future studies could further test these ideas in more naturalistic settings, for example, using music or speech stimuli, which incorporate predictions at different hierarchical levels.

Another novelty in our paradigm was the introduction of a low-level manipulation in addition to the abstract rule manipulation. To do so, we created stimuli in which a change occurred in a local feature, such that the phase was flipped within each tone in the sequence in some blocks but not in others. In doing so, we could orthogonally test whether the observed effects were high-level specific (i.e. related to sound patterns) or if on the contrary similar dynamics could be observed as a function of low-level features related to stimulus periodicity that do not require sensitivity to how the current tone relates to prior tones. As opposed to the results of the oddball design, we did not observe effects associated with predictability in low-level features (Fig. 8). This suggests that the phenomena under study were high-level specific and not elicited by just any type of acoustic regularity. As a note of caution, however, we must point out the possibility that the BOLD signal measured in the present work only captures aggregated effects such as those observed in the AC to the presence of a noise disruption (Fig. 8) and is not responsive to the finer-grained neural dynamics associated with phase perturbation, since hemodynamic responses are much too slow to be sensitive to sub-ms signal changes. In order to study whether there are neural dynamics indicative of predictability in local features, future studies using techniques with higher temporal resolution, such as magnetoencephalography (MEG) or intracranial electroencephalography (EEG), which can be used to record low-level correlates of sound representation such as periodicity encoding (e.g. Coffey et al. 2019) should be used.

The current data do not support the conclusion that subcortical and cortical structures demonstrate prediction-based inhibition similarly but independently from one another, even though sensitivity to pattern-based prediction could be detected at both cortical and subcortical levels, in accord with prior studies (Tabas and von Kriegsten 2024). Rather, it seems more likely that the modulation of subcortical signals associated with the detection of auditory deviants based on pattern-level information is computed in and propagated from the auditory cortex. This conclusion fits well with nonhuman physiological studies that have dissociated sensitivity to lower-level features from processing based on more abstract properties of the stimulus (Parras et al. 2017). It also is consistent with more general considerations about hierarchical organization of perceptual processes proceeding from more stimulus-driven responses at earlier levels toward more cognitive-driven representations at later stages (Li et al. 2023; Wolff et al. 2022).

## Supplementary Material

Supplementary_materials_bhae316
