# Supplementary material for: Cortical–subcortical interactions underlie processing of auditory predictions measured with 7T fMRI
Source: Cereb Cortex. 2024 Aug 1;34(8):bhae316. doi: 10.1093/cercor/bhae316 (PMC11292673; doi:10.1093/cercor/bhae316)
Supplement: Supplementary_materials_bhae316 [file supplementary_materials_bhae316.pdf]

Cortical-subcortical interactions underlie processing of auditory predictions measured with 7T fMRI: supplementary materials

Alberto Ara, Vasiliki Provias, Kevin Sitek, Emily B.J. Coffey, Robert J. Zatorre

3-alternative forced choice signal detection theory model

In order to analyze the behavioral data from the oddball task, a 3-alternative forced choice (3-AFC) signal detection theory (SDT) model was used. According to this model, the likelihood that the observer will correctly identify the target stimulus upon perceiving all three intervals depends on their ability to discriminate between signals, or d' (DeCarlo, 2012). In this case, it is assumed that the observer chooses the position with the largest perceived magnitude as the signal. If the observer's discriminability is good enough, the target will be correctly identified in the interval with the strongest percept. If, however, the observer's discriminability is low, another position might be perceived as stronger due to the magnitude of the noise, generating incorrect responses.

The estimation of d' can be thought of a mixture model where each observation i from participant j is drawn from two Bernoulli distributions. In the first Bernoulli process, we model with likelihood θ_{1ij} that position 1 is chosen over positions 2 and 3 (i.e. $y_{1ij} = 1$). In the second Bernoulli process, we model with likelihood θ_{2ij} that position 2 is chosen over positions 1 and 3 (i.e. $y_{2ij} = 1$):

$$y_{1ij} \sim Bernoulli(\theta_{1ij})$$

$$y_{2ij} \sim Bernoulli(\theta_{2ij})$$

θ_{1ij} and θ_{2ij} are in turn given by the SDT model for the 3-AFC task:

$$\theta_{1ij} = \int_{-\infty}^{\infty} \Phi\left(d'_j Z_{ij} + e_{1ij}\right) \Phi\left(-d'_j Z_{1ij} + e_{1ij}\right) \varphi\left(e_{1ij}\right) d'e_{1ij}$$

$$\theta_{2ij} = \int_{-\infty}^{\infty} \Phi\left(-d'_j Z_{ij} + e_{2ij}\right) \Phi\left(-d'_j Z_{2ij} + e_{2ij}\right) \varphi\left(e_{2ij}\right) d'e_{2ij}$$

with $Z_{ij} = X_{1ij} - X_{2ij}$; $Z_{1ij} = 1 - 2X_{1ij} - X_{2ij}$; $Z_{2ij} = 1 - X_{1ij} - 2X_{2ij}$; and X_{1ij} and X_{2ij} are coding whether the signal is in position 1 or 2 (or none), respectively (i.e. position 1: $X_{1ij} = 1$, $X_{2ij} = 0$; position 2: $X_{1ij} = 0$, $X_{2ij} = 1$; position 3: $X_{1ij} = 0$, $X_{2ij} = 0$). e_{1ij} and e_{2ij} represent random variation, or noise, in the perception for each Bernoulli process. d' is shared across both Bernoulli processes, and is defined by an unknown grand mean α and individual deflections of participant ν_j, the latter drawn from a normal distribution centered at zero and unknown hierarchical standard deviation σ:

$$d'_j = \alpha + \nu_j$$

$$\nu_j \sim Normal(0, \sigma)$$

Finally, the unknown parameters of the mixture model are estimated from the data using approximation and assuming weakly informative prior distributions. We chose weakly informative prior distributions as an agnostic enough choice that would nonetheless properly define the parameter space:

$$\alpha \sim Normal(0,1)$$

$$\sigma \sim Gamma(1,1)$$

$$e_{1ij}, e_{2ij} \sim Normal(0,1)$$

1st level analysis of brain data

In order to obtain each participant's and ROI's neural response to each condition of interest the mean estimates and their associated measurement error (i.e. standard error of the estimate) were extracted using SPM and MarsBar (Penny et al., 2011; Brett et al., 2002). For simplicity, we used the classical restricted maximum likelihood estimation procedure to estimate the model parameters. In addition to the conditions and regressors in each model, other details include timing parameters and specific transformations to the data.

Regarding timing parameters, the units per design and onsets were specified in seconds with an interscan interval set to the repetition time of the image acquisition (TR = 1.53). The micro-time resolution (i.e. the number of bins with which each scan is sampled when convolving regressors) was set to the number of slices per scan (nSlices = 48); and the micro-time onset (i.e. the reference time-bin to which the regressors are offset) was set to the first slice of each scan (i.e. no offset).

Regarding data transformations, the default SPM high-pass filter of 128 seconds and auto-correlation whitening of order 1 -AR(1)- were applied. Regressors were convolved using SPM's canonic hemodynamic response function. Each ROI time course y was estimated separately with global normalization across session. This type of scaling maximizes sensitivity by multiplying each signal value Y at time t in session s by the constant value 100 divided by the average amplitude of that session μ (Andersson et al., 2001):

$$\hat{y}_{ts} = y_{ts}\frac{100}{\mu_s}$$

Once the model parameters were estimated, contrasts were defined for each condition of interest in all sessions with a contrast weight of 1/nSession and every other condition zeroed out. We then finally extracted from MarsBar each condition's mean estimate and their associated measurement error, computing the latter by dividing the mean estimates by their corresponding t-values returned by MarsBar.

2st level analysis of brain data

In order to analyze the data at the group level while preserving the precision of the 1st level analyses we used a mixed-effects multilevel analysis (MEMA). This is a sensible improvement from the classical two-stage analysis, where 1st level estimates are reduced to summary statistics and are ultimately treated as mere observations in a classical test. While this approach is simple and widely used, it comes at the expense of losing valuable information. This is especially critical in designs studying basic physiological phenomena (e.g. neural responses to stimuli), where the statistical precision depends on the number of trials, and each participant is considered, put simply, a replication of the same experiment. In such cases, it is clear to see how such an experiment with a priori sufficient observations (e.g. nTrials = 216 per nSubjects = 10) gets reduced to an insufficient sample size if the classical two-step approach is employed. On the other hand, performing inferences at every individual level separately could incur in atomistic fallacies, in addition to offering an inelegant solution. A popular choice in such cases is a MEMA. This

method allows to preserve the 1st levels' precision while still drawing inferences at the group level in aggregate, thus maximizing statistical power (Chen et al., 2012).

While the most widely used approach for MEMA is full hierarchical models -where every observation from every individual is pooled- this is a computationally expensive approach in the case of fMRI analysis, given the complexity of the models. An alternative approach with very similar results is to still analyze the data in two stages but incorporating the measurement error of the 1st level estimates into the 2nd level (Chen et al., 2012). This is achieved by estimating meta-analytically the "true" group-level effect from each individual observed effect. In our case, each observed effect y comes from a given condition i and a given participant j. Since we have four dependent variables (i.e. ROIs), we model them as correlated with a multivariate gaussian likelihood function:

$$[y_{1ij} \quad y_{2ij} \quad y_{3ij} \quad y_{4ij}] = \mathcal{N}(\mu_{ij}, \Sigma_{ij})$$

Where μ_{ij} is a vector of true population means (for all four ROIs) and $\boldsymbol{\Sigma}_{ij}$ a covariance matrix:

$$\mu_{ij} = \begin{bmatrix} \theta_{1ij} \\ \theta_{2ij} \\ \theta_{3ij} \\ \theta_{4ij} \end{bmatrix}$$

$$\Sigma_{ij} = \begin{bmatrix} \sigma_{1ij}^2 & & & \\ \rho_{12}\sigma_{1ij}\sigma_{2ij} & \sigma_{2ij}^2 & & \\ \rho_{13}\sigma_{1ij}\sigma_{3ij} & \rho_{23}\sigma_{2ij}\sigma_{3ij} & \sigma_{3ij}^2 & \\ \rho_{14}\sigma_{1ij}\sigma_{4ij} & \rho_{24}\sigma_{2ij}\sigma_{4ij} & \rho_{34}\sigma_{3ij}\sigma_{4ij} & \sigma_{4ij}^2 \end{bmatrix}$$

With each ρ quantifying the unknown correlation between two given outcomes (i.e. ROIs) and each σ_{ij} pooling the unknown population error τ with each measurement error ME_{ij}:

$$\sigma_{1ij} = \sqrt{\tau_1^2 + ME_{1ij}^2};\ \sigma_{2ij} = \sqrt{\tau_2^2 + ME_{2ij}^2}; \sigma_{3ij} = \sqrt{\tau_3^2 + ME_{3ij}^2}; \sigma_{4ij} = \sqrt{\tau_4^2 + ME_{4ij}^2}$$

In turn, and since we are modeling repeated measures (i.e. experimental conditions), each population mean θ_{ij} is defined by an unknown grand mean α and individual deflections of experimental condition ν_i and participant ν_j:

$$\theta_{1ij} = \alpha_1 + \nu_{1i} + \nu_{1j}; \theta_{2ij} = \alpha_2 + \nu_{2i} + \nu_{2j}; \theta_{3ij} = \alpha_3 + \nu_{3i} + \nu_{3j}; \theta_{4ij} = \alpha_4 + \nu_{4i} + \nu_{4j}$$

The latter drawn from a normal distribution centered at zero and unknown hierarchical standard deviations ζ and γ:

$$\nu_{1i}, \sim Normal(0, \zeta_1); \nu_{2i} \sim Normal(0, \zeta_2); \nu_{3i} \sim Normal(0, \zeta_3); \nu_{4i} \sim Normal(0, \zeta_4)$$

$$\nu_{1j}, \sim Normal(0, \gamma_1); \nu_{2j} \sim Normal(0, \gamma_2); \nu_{3j} \sim Normal(0, \gamma_3); \nu_{4j} \sim Normal(0, \gamma_4)$$

Finally, the unknown parameters are estimated from the data using approximation and assuming weakly informative prior distributions:

$$\tau_1, \tau_2, \tau_3, \tau_4 \sim Gamma(1,1)$$

$$\alpha_1, \alpha_2, \alpha_3, \alpha_4 \sim Normal(0,1)$$

$$\zeta_1, \zeta_2, \zeta_3, \zeta_4 \sim Gamma(1,1)$$

$$\gamma_1, \gamma_2, \gamma_3, \gamma_4 \sim Gamma(1,1)$$

$$\begin{bmatrix} \rho_{12} & & \\ \rho_{13} & \rho_{23} & \\ \rho_{14} & \rho_{24} & \rho_{34} \end{bmatrix} \sim LKJ(3)$$

Effective connectivity analysis of initial tone detection

In order to assess the directionality of initial tone detection along the auditory pathway we estimated and compared two path analyses (McIntosh and Gonzalez-Lima, 1994) with opposing directions. These models were fit on the first tone data only (i.e. std0). These models consisted of four independent regression lines where the average neural response to the stimulus was estimated for a given ROI from activity in the preceding ROI, or its average activity in the case of the first path in the model. Similar to a MEMA, since the 1st level estimates error was observed, we fitted the models with measurement error for both responses and predictors (McElreath, 2020). In both cases, we first modeled the neural response per participant j in ROI 1 with Gaussian likelihood:

$$y_{1j} = \mathcal{N}(\theta_1, \sigma_{1j})$$

Where the population mean θ_1 is given by the unknown average α_1:

$$\theta_1 = \alpha_1$$

And standard deviation σ_{1j} pools the unknown population error τ_1 with each measurement error ME_{1j}:

$$\sigma_{1j} = \sqrt{\tau_1^2 + ME_{1j}^2}$$

Next in the path, the neural response in ROI 2 y_{2j} is modeled with measurement error ME_{2j}:

$$y_{2j} \sim \mathcal{N}(\theta_{2j}, \sigma_{2j})$$

$$\theta_{2j} = \alpha_2 + \beta_1 X_{1j}$$

$$\sigma_{2j} = \sqrt{\tau_2^2 + ME_{2j}^2}$$

Where β_1 is the unknown weight of the slope and the predictor X_{1j} represents the "true" neural response in ROI 1, which is found by modeling the observed neural response x_{1j} with measurement error ME_{1j} with Gaussian likelihood:

$$x_{1j} \sim \mathcal{N}(X_{1j}, ME_{1j})$$

Likewise, in the remaining two paths the neural response in ROIs 3 and 4 are predicted from the true neural response in ROIs 2 and 3 with measurement error, respectively:

$$y_{3j} \sim \mathcal{N}(\theta_{3j}, \sigma_{3j}); y_{4j} \sim \mathcal{N}(\theta_{4j}, \sigma_{4j})$$

$$\sigma_{3j} = \sqrt{\tau_3^2 + ME_{3j}^2};\ \sigma_{4j} = \sqrt{\tau_4^2 + ME_{4j}^2}$$

$$\theta_{3j} = \alpha_3 + \beta_2 X_{2j};\ \theta_{4j} = \alpha_4 + \beta_3 X_{3j}$$

$$x_{2j} \sim \mathcal{N}(X_{2j}, ME_{2j});\ x_{3j} \sim \mathcal{N}(X_{3j}, ME_{3j})$$

Since the true predictor values X_{1j}, X_{2j} and X_{3j} are internally handled as latent variables, these are drawn from a multivariate normal distribution with unknown means μ, standard deviations γ and correlation ρ:

$$[X_{1j} \quad X_{2j} \quad X_{3j}] \sim MVNormal(\mu, \Sigma)$$

$$\mu = \begin{bmatrix} \chi_1 \\ \chi_2 \\ \chi_3 \end{bmatrix}$$

$$\Sigma = \begin{bmatrix} \gamma_1^2 & & \\ \rho_{12}\gamma_1\gamma_2 & \gamma_2^2 & \\ \rho_{13}\gamma_1\gamma_3 & \rho_{23}\gamma_2\gamma_3 & \gamma_3^2 \end{bmatrix}$$

Finally, the unknown parameters are estimated from the data using approximation and assuming weakly informative prior distributions:

$$\tau_1, \tau_2, \tau_3, \tau_4 \sim Gamma(1,1)$$

$$\alpha_1, \alpha_2, \alpha_3, \alpha_4 \sim Normal(0,1)$$

$$\beta_1, \beta_2, \beta_3 \sim Normal(0,1)$$

$$\chi_1, \chi_2, \chi_3 \sim Normal(0,1)$$

$$\gamma_1, \gamma_2, \gamma_3 \sim Gamma(1,1)$$

$$\begin{bmatrix} \rho_{12} & \\ \rho_{13} & \rho_{23} \end{bmatrix} \sim LKJ(3)$$

Conceptually, the relation between outcomes and predictors in the two competing models are:

- Bottom-up model:
  - 1st path: Average IC activity
  - 2nd path: IC activity → MGB activity
  - 3rd path: MGB activity → AC activity
  - 4th path: AC activity → STG activity
- Top-down model:
  - 1st path: Average STG activity
  - 2nd path: STG activity → AC activity
  - 3rd path: AC activity → MGB activity
  - 4th path: MGB activity → IC activity

The intercept only model consisted in the same set of regressions, but all ROIs activity were predicted from their average only, zeroing out all βY_i terms:

- Intercept-only model:

- 1st path: Average IC activity
- 2nd path: Average MGB activity
- 3rd path: Average AC activity
- 4th path: Average STG activity

Effective connectivity analysis of predictive suppression

In order to assess the directionality of the deviant position effect along those ROIs showing predictive suppression we estimated and compared two path analyses with opposing directions. These models were fit on the deviant data only, after standardizing observations per participant and ROI. These models consisted of three independent regression lines with measurement error where the neural response to the stimulus was estimated for a given ROI from activity in the preceding ROI, or the position of the deviant in the case of the first path in the model. In both cases, we first modeled the neural response i per participant j in ROI 1 with Gaussian likelihood:

$$y_{1ij} = \mathcal{N}(\theta_{1ij}, \sigma_{1ij})$$

Where the population mean θ_{1ij} is given by the following regression equation:

$$\theta_{1ij} = \alpha_1 + \nu_{1j} + \beta_0 X_{0ij}$$

And the standard deviation σ_{1ij} pools the unknown population error τ_1 with each measurement error ME_{1ij}:

$$\sigma_{1ij} = \sqrt{\tau_1^2 + ME_{1ij}^2}$$

In the regression equation, α_1 is the unknown intercept, X_{0ij} is the deviant position, β_0 is the unknown weight of the slope, and ν_{1j} accounts for varying intercepts per participant, the latter drawn from a normal distribution centered at zero with unknown hierarchical standard deviation ζ_1:

$$\nu_{1j} \sim Normal(0, \zeta_1)$$

Next in the path, the neural response in ROI 2 y_{2ij} is also modeled with measurement error ME_{2ij}:

$$y_{2ij} \sim \mathcal{N}(\theta_{2ij}, \sigma_{2ij})$$

$$\theta_{2ij} = \alpha_2 + \nu_{2j} + \beta_1 X_{1ij}$$

$$\sigma_{2ij} = \sqrt{\tau_2^2 + ME_{2ij}^2}$$

$$\nu_{2j} \sim Normal(0, \zeta_2)$$

Where the predictor X_{1ij} represents the "true" neural response in ROI 1, which is found by modeling the observed neural response x_{1ij} with measurement error ME_{1ij} with Gaussian likelihood:

$$x_{1ij} \sim \mathcal{N}(X_{1ij}, ME_{1ij})$$

Likewise, in the remaining path the neural response in ROIs 3 is predicted from the true neural response in ROIs 2 with measurement error, respectively:

$$y_{3ij} \sim \mathcal{N}(\theta_{3ij}, \sigma_{3ij})$$

$$\sigma_{3ij} = \sqrt{\tau_3^2 + ME_{3ij}^2}$$

$$\theta_{3ij} = \alpha_3 + \nu_{3j} + \beta_2 X_{2ij}$$

$$\nu_{3j} \sim Normal(0, \zeta_3)$$

$$x_{2ij} \sim \mathcal{N}(X_{2ij}, ME_{2ij})$$

Since the true predictor values X_{1ij} and X_{2ij} are internally handled as latent variables, these are drawn from a multivariate normal distribution with unknown means μ, standard deviation γ and correlation ρ:

$$[X_{1ij} \quad X_{2ij}] \sim MVNormal(\mu, \Sigma)$$

$$\mu = \begin{bmatrix} \chi_1 \\ \chi_2 \end{bmatrix}$$

$$\Sigma = \begin{bmatrix} \gamma_1^2 & \\ \rho_{12}\gamma_1\gamma_2 & \gamma_2^2 \end{bmatrix}$$

Finally, the unknown parameters are estimated from the data using approximation and assuming weakly informative prior distributions:

$$\tau_1, \tau_2, \tau_3 \sim Gamma(1,1)$$

$$\alpha_1, \alpha_2, \alpha_3 \sim Normal(0,1)$$

$$\zeta_1, \zeta_2, \zeta_3 \sim Gamma(1,1)$$

$$\beta_1, \beta_2, \beta_3 \sim Normal(0,1)$$

$$\chi_1, \chi_2 \sim Normal(0,1)$$

$$\gamma_1, \gamma_2 \sim Gamma(1,1)$$

$$\rho_{12} \sim LKJ(3)$$

Conceptually, the relation between outcomes and predictors in the two competing models are:

- Bottom-up model:
  - 1st path: Deviant position → IC activity
  - 2nd path: IC activity → MGB activity
  - 3rd path: MGB activity → AC activity
- Top-down model:
  - 1st path: Deviant position → AC activity
  - 2nd path: AC activity → MGB activity
  - 3rd path: MGB activity → IC activity

The intercept only model consisted in the same set of regressions, but all ROIs activity were predicted from their average only (plus per-participant deflections from average), zeroing out all βY_i terms:

- Intercept-only model:
  - 1st path: Average IC activity

- 2nd path: Average MGB activity
- 3rd path: Average AC activity

References

- Andersson JLR, Ashburner J, Friston K (2001) A global estimator unbiased by local changes. Neuroimage 13(6):1193-1206.
- Brett M, Anton J-L, Valabregue R, Poline J-B, Others (2002) Region of interest analysis using an SPM toolbox. In: 8th international conference on functional mapping of the human brain, pp 497. Sendai.
- Chen G, Saad ZS, Nath AR, Beauchamp MS, Cox RW (2012) FMRI group analysis combining effect estimates and their variances. Neuroimage 60:747–765.
- DeCarlo LT (2012) On a signal detection approach to m-alternative forced choice with bias, with maximum likelihood and Bayesian approaches to estimation. J Math Psychol 56(3):196-207.
- Kruschke J (2015) Doing Bayesian data analysis: A tutorial with R, JAGS, and Stan. Elsevier.
- Makowski D, Ben-Shachar MS, Chen SHA, Lüdecke D (2019) Indices of Effect Existence and Significance in the Bayesian Framework. Front Psychol 10:2767.
- McElreath R (2018) Statistical rethinking: A Bayesian course with examples in R and Stan. Chapman and Hall/CRC.
- McIntosh AR, Gonzalez-Lima F (1994) Structural modeling of functional neural pathways mapped with 2-deoxyglucose: effects of acoustic startle habituation on the auditory system. Brain Res 547:295–302.
- Penny WD, Friston KJ, Ashburner JT, Kiebel SJ, Nichols TE (2011) Statistical Parametric Mapping: The Analysis of Functional Brain Images. Elsevier.
- Watanabe S, Opper M (2010) Asymptotic equivalence of Bayes cross validation and widely applicable information criterion in singular learning theory. J Mach Learn Res.
